# Direct-to-consumer marketing (DTC) of internet-based cognitive behavioral therapy (iCBT) using brief promotional video: Mapping modifiable mechanisms in pre-treatment acceptance

**DOI:** 10.1192/j.eurpsy.2021.1846

**Published:** 2021-08-13

**Authors:** L. Auyeung, E. Wong, W. Mak

**Affiliations:** 1 Department Of Psychology, The Chinese University of Hong Kong, Sha Tin, Hong Kong PRC; 2 Psychology, The Chinese University of Hong Kong, Shatin, Hong Kong PRC

**Keywords:** Direct-to-consumer Marketing, Internet-based Cognitive Behavioral Therapy, The Unified Theory of Acceptance and Use of Technology, Depression

## Abstract

**Introduction:**

Given the suboptimal acceptance of iCBT for depression, finding ways to increase the acceptance and uptake is crucial for its dissemination. Moreover, it remains unknown to what extend the Unified Theory of Acceptance and Use of Technology (UTAUT) could aid the design of DTC in psychological service.

**Objectives:**

To explore whether the regulatory processes theorized in the UTAUT (Performance Expectancy, Effort Expectancy, Social Influence, and Facilitating Condition) could be modified and mediate the change of acceptance of iCBT.

**Methods:**

This randomized controlled trial recruited 219 individuals with at least mild level of depression. Upon completion of pre-assessment, participants were randomly allocated to an intervention (IG) and a control group (CG). The IG received a 7-minute UTAUT-driven promotion video, while the CG received a video of same length on general psychoeducation. Both groups completed a post-assessment.

**Results:**

Repeated measures ANOVA revealed a significant time by group effect on treatment acceptance. The video in IG was perceived to be clearer and more persuasive. Mediation analysis showed that the intervention effect was mediated by increase in perceived performance expectancy of iCBT, and the indirect effect was conditional on dispositional help seeking stigma.

**Conclusions:**

Pre-treatment acceptance of iCBT can be improved by brief DTC promotion video. The finding casts light that performance expectancy was the most modifiable and mediatable regulatory process on iCBT acceptance, although such relation could be attenuated by high help-seeking stigma. In sum, DTC marketing could aid implementation of Internet-based interventions, effort in stigma reduction should continue to encourage uptake of effective treatment.

**Disclosure:**

No significant relationships.

